# Interspecies Metabolic Complementation in Cystic Fibrosis Pathogens via Purine Exchange

**DOI:** 10.3390/pathogens10020146

**Published:** 2021-02-01

**Authors:** Hafij Al Mahmud, Jiwasmika Baishya, Catherine A. Wakeman

**Affiliations:** Department of Biological Sciences, Texas Tech University, Lubbock, TX 2901, USA; hafij-al.mahmud@ttu.edu (H.A.M.); Jiwasmika.baishya@ttu.edu (J.B.)

**Keywords:** auxotrophy, purine, cystic fibrosis infection, polymicrobial interactions, cross feeding, *Pseudomonas aeruginosa*, *Staphylococcus aureus*

## Abstract

Cystic fibrosis (CF) is a genetic disease frequently associated with chronic lung infections caused by a consortium of pathogens. It is common for auxotrophy (the inability to biosynthesize certain essential metabolites) to develop in clinical isolates of the dominant CF pathogen *Pseudomonas aeruginosa*, indicating that the CF lung environment is replete in various nutrients. Many of these nutrients are likely to come from the host tissues, but some may come from the surrounding polymicrobial community within the lungs of CF patients as well. To assess the feasibility of nutrient exchange within the polymicrobial community of the CF lung, we selected *P. aeruginosa* and *Staphylococcus aureus*, two of the most prevalent species found in the CF lung environment. By comparing the polymicrobial culture of wild-type strains relative to their purine auxotrophic counterparts, we were able to observe metabolic complementation occurring in both *P. aeruginosa* and *S. aureus* when grown with a purine-producing cross-species pair. While our data indicate that some of this complementation is likely derived from extracellular DNA freed by lysis of *S. aureus* by the highly competitive *P. aeruginosa*, the partial complementation of *S. aureus* purine deficiency by *P. aeruginosa* demonstrates that bidirectional nutrient exchange between these classic competitors is possible.

## 1. Introduction

Cystic fibrosis (CF) is an autosomal recessive disorder caused by mutations in the cystic fibrosis transmembrane conductance regulator (CFTR) gene, but the primary reason for patient morbidity and mortality is often associated with pulmonary infection [1]. *Pseudomonas aeruginosa*, *Staphylococcus aureus*, *Haemophilus influenzae*, and *Burkholderia* sp. are some of the major bacterial pathogens associated with CF infection. The complex interplay between these organisms plays a vital role in disease progression and pathogenesis [2,3]. Furthermore, other biological factors, including host-derived immune responses and interspecies interactions, help determine the overall composition and structure of the microbial community causing severe infection and contributing to the outcome of this disorder [2].

The major pathogen *P. aeruginosa* has been shown to exhibit multiple auxotrophies in CF lungs [4,5]. High levels of amino acid concentration in the infected lung airways can play an important role in selecting and maintaining some of these biosynthetically-deficient strains [6]. Interestingly, certain *P. aeruginosa* strains from CF communities can also crossfeed each other amino acids to complement these metabolic deficiencies emerging in the CF lung environment, indicating that intraspecies nutrient exchange can be an additional factor in the selection for auxotrophy during chronic infection [7,8]. Both intra- and interspecies nutrient exchange in the context of the CF polymicrobial consortium appear to be possible since metabolic deficiencies, such as loss of heme and menaquinone biosynthesis that causes the emergence of non-respiring small colony variants, can be rescued via cross feeding in both laboratory and clinical isolates [9]. This in vitro phenomenon may be occurring in vivo due to the fact that different species such as *P. aeruginosa* and *S. aureus* have previously been found to occupy the same airspaces within the CF lung [10]. Therefore, we hypothesize that the polymicrobial consortium is a source of metabolites in the CF environment that may contribute to the emergence of auxotrophic strains during chronic infection. 

An analysis of the essential genes of *P. aeruginosa* grown in sputum media showed that purines, along with 45 other metabolites, are molecules that are likely to be critical during late-stage infection due to their minimal bioavailability in the CF lung environment [11]. Purines are essential for numerous cellular activities in *P. aeruginosa* and hence could be a possible metabolite that could be exchanged between CF pathogens. The deoxy form of purine diphosphate derivatives is used as a precursor for DNA synthesis, and purine monophosphates are synthesized newly by de novo synthesis or recycled from nucleic acid turnover by salvage pathway [12]. In *P. aeruginosa*, inosine monophosphate (IMP), the precursor in purine biosynthesis, can be synthesized from 5-phosphoribosyl-1-pyrophosphate (PRPP) by a de novo biosynthesis pathway involving 11 enzymatic steps. These essential enzymes are encoded by different genes such as *purC*, *purD*, *purK*, etc. Finally, adenosine- and guanosine-monophosphates can be synthesized from IMP separately [13,14]. In this study, we have conducted co-culture experiments with purine auxotrophic strains to investigate whether purine deficiency can be bi-directionally exchanged between *P. aeruginosa* and *S. aureus* as proof-of-principle that metabolic complementation can occur between these classic competitors.

## 2. Results and Discussion

### 2.1. The Growth of a Purine-Deficient Mutant of P. aeruginosa Can Be Complemented by the Presence of S. aureus

**Figure 1:**
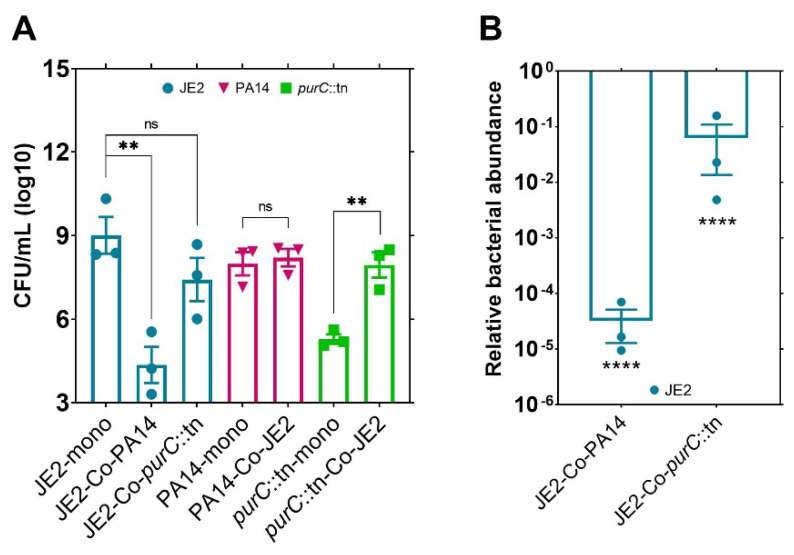
Growth of a purine-deficient mutant of *P. aeruginosa* can be rescued in the presence of *S. aureus*. (**A**) Purine complementation in purine-deficient mutant (*purC*::tn) of *P. aeruginosa* by an expected/a probable purine-producing strain of *S. aureus* (JE2) in co-culture. (**B**) Relative bacterial abundance of JE2 in co-culture with PA14 or *purC*::tn mutant strain of *P. aeruginosa* compared to JE2 monoculture. Error bars represent SEM of data derived from three biological replicates on different days, and experiments were performed in technical triplicates each day. Here, ‘’**’’ designates *p* < 0.005, ‘’****’’ designates *p* < 0.0001 as depicted by two-tailed unpaired Student’s *t*-test, and ns denotes not significant (*p* > 0.05).

In this study, we sought to explore possible nutrient exchanges between auxotrophic strains of *P. aeruginosa* and *S. aureus*. Specifically, we were interested in an exchange of purines since they are essential for growth of *P. aeruginosa* in CF lungs as well as for evading host immunity [11,15]. We first performed experiments to determine if a transposon mutant strain of *P. aeruginosa* deficient in purine biosynthesis (*purC*::tn) could be complemented to growth levels of its parental strain (PA14) by the presence of a purine-producing strain of *S. aureus* (JE2) when cultured in purine-deficient growth media. Indeed, upon co-culture with JE2, growth of this mutant was rescued significantly, presumably via complementation of purines (Figure 1A). However, in co-culture with wild-type *P. aeruginosa* (PA14), JE2 cells were being killed, a typical anti-staphylococcal behavior shown by most strains of *P. aeruginosa* [16]. Wild-type *P. aeruginosa* cells were more aggressive in killing JE2 compared to *purC*::tn. A possible explanation behind this altered phenotype could be that purine auxotrophy led to a reduction in the pathogenicity or competitive phenotype of the purine-deficient strain of *P. aeruginosa*. Growth retardation and weak virulence due to purine auxotrophy have also been observed in other bacteria such as *S. aureus* [17], *Escherichia coli* [18], *Listeria monocytogenes* [19], *Bacillus anthracis* [20], and *Brucella abortus* [21], supporting the possible role of purines in reduced competitiveness in *P. aeruginosa purC*::tn as well. In addition to this, the density of JE2 did not change significantly in co-culture with *purC*::tn versus its monoculture (Figure 1B), which indicates that purines shared via cross feeding to *P. aeruginosa purC*::tn may not simply be sourced from cellular degradation of JE2. Hence, this study supports that the growth of purine-deficient mutant of *P. aeruginosa* can be rescued significantly by *S. aureus* through purine cross-feeding.

### 2.2. Exogenous DNA Complements the Growth of P. aeruginosa and May Contribute to the Rescue of Purine-Deficient P. aeruginosa by S. aureus

**Figure 2:**
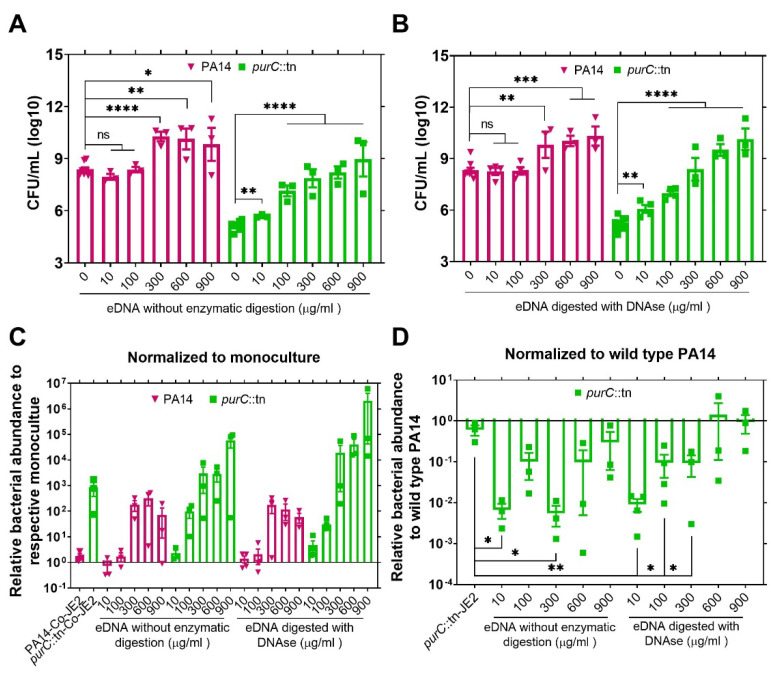
Exogenous DNA provides a nutrient source for *P. aeruginosa* and can rescue the growth of a purine-deficient mutant. (**A**) Bacterial cells were incubated with 10 to 900 µg/mL herring DNA, as an exogenous purine source, without enzymatic digestion. (**B**) Bacterial cells were incubated with 10 to 900 µg/mL herring DNA with enzymatic digestion. (**C**) Relative bacterial abundance of wild-type and purine-deficient mutant (*purC*::tn) of *P. aeruginosa* in co-culture with JE2 or exogenous DNA. Cells in co-culture with JE2 or eDNA were normalized to the respective numbers in monoculture. (**D**) Relative fitness of purine-deficient mutant (*purC*::tn) of *P. aeruginosa* in co-culture with JE2 or eDNA compared to the growth of wildtype *P. aeruginosa*, PA14, in co-culture with JE2 or eDNA, respectively. Here, the relative numbers of *purC*::tn mutant were calculated by normalizing to the wild-type PA numbers in respective co-culture conditions. Error bars represent SEM of data derived from at least three biological replicates on different days, and experiments were performed in technical triplicates each day. The statistical comparison was done between the *purC*::tn mutant and PA14 strains (not shown here) in their respective co-culture conditions. Here, ‘’*’’ designates *p* < 0.05, ‘’**’’ designates *p* < 0.005, ‘’***’’ designates *p* < 0.0005, ‘’****’’ designates *p* < 0.0001 depicted by two-tailed unpaired Student’s *t*-test, and ns denotes not significant. The concentration of the exogenous DNA is in µg/mL.

After demonstrating that purine-deficient mutant of *P. aeruginosa* can be rescued by *S. aureus* in co-culture, we wanted to determine whether the growth of *purC*::tn can be rescued by exogenous DNA (eDNA). It is still possible that eDNA may be released into the media by lysed *S. aureus* even though our data indicate that *S. aureus* death in the presence of *purC*::tn is significantly reduced compared to the cell death that occurs in the presence of wild-type *P. aeruginosa*. Another reason to assess the complementation of *purC*::tn by eDNA is that multiple studies have shown that chronic infections of the CF airways are frequently associated with biofilms containing large amounts of eDNA [22,23]. The eDNA may be sourced from dead microbial cells or from host innate immune components, such as lysed polymorphonuclear leukocytes and neutrophil extracellular traps [24,25,26]. A study of 132 CF patients reported that the concentration of eDNA in sputum could vary from 0 to 900 µg/mL across different CF patients [27]. Therefore, in this experiment, we tested if enzymatically digested and undigested eDNA, at concentrations ranging from 10 to 900 µg/mL, can rescue the growth of *P. aeruginosa purC*::tn cells and found that eDNA supplementation to the media increases the growth of both wild-type PA14 and the *purC*::tn mutant with or without enzymatic digestion (Figure 2A,B). In co-culture with digested or undigested eDNA, this growth increase in both the wild-type and mutant strains of *P. aeruginosa* is even more apparent when the data are normalized relative to monoculture-colony-forming units (CFUs) (Figure 2C). When normalized relative to wild-type PA14 growth levels in respective bacterial or eDNA co-culture, complete rescue of the *purC*::tn growth by eDNA occurs in eDNA co-culture at eDNA levels exceeding 300 µg/mL (Figure 2D). The average relative fitness of *purC*::tn showed a gradual increase with the increasing concentration of enzymatically digested eDNA. Non-digested eDNA also showed a similar trend with an aberrant decrease in the fitness of *purC*::tn for the concentration of 300 µg/mL compared to 100 µg/mL. This disproportionate average fitness could have resulted from the differential growth dynamics of PA14 to *purC*::tn with eDNA.

**Figure 3:**
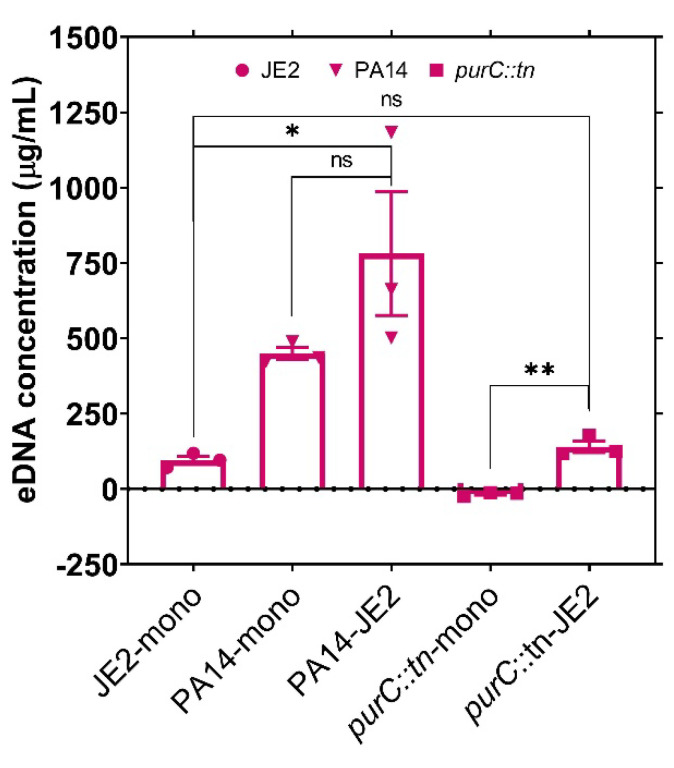
The concentration of exogenous DNA in culture medium after bacterial growth. Bacterial cells were grown as monoculture or co-culture in RPMI (Roswell Park Memorial Institute) media. The concentration (µg/mL) of exogenous DNA was measured by PicoGreen dsDNA (double stranded DNA) reagent following 48 h of incubation. Error bars represent SEM of data derived from three biological replicates on different days, and experiments were performed in technical triplicates each day. Here, ‘’*’’ designates *p* < 0.05, ‘’**’’ designates *p* < 0.005 as depicted by two-tailed unpaired Student’s *t*-test, and ns denotes not significant.

In order to determine if the rescue of *P. aeruginosa purC*::tn by *S. aureus* is mediated by eDNA released during culture either by *P. aeruginosa*-mediated lysis or by autolysis, we measured the levels of eDNA present in the growth medium in both mono- and co-culture. While co-culture in the presence of wild-type PA14 resulted in higher levels of eDNA release as expected due to the known antistaphylococcal capabilities of *P. aeruginosa*, growth in the presence of the *purC*::tn mutant did not significantly increase the levels of eDNA released relative to *S. aureus* JE2 monoculture (Figure 3). Even in monoculture, there was still a significant level of eDNA accumulation (~100 µg/mL) in *S. aureus* cultures likely deriving from known autolytic mechanisms [28,29,30,31]. However, the eDNA values in *S. aureus* monoculture or the co-culture with *purC*::tn were significantly lower than the >300 µg/mL shown in Figure 2D required for complete rescue of the *purC*::tn mutant. Therefore, while it is possible that cell lysis is contributing to the co-culture mediated rescue of the *purC*::tn mutant, it is likely that much of the rescue effect can be attributed to the natural metabolism of *S. aureus.*

### 2.3. The Growth of a Purine-Deficient Mutant of S. aureus Can Be Complemented by the Presence of P. aeruginosa

**Figure 4:**
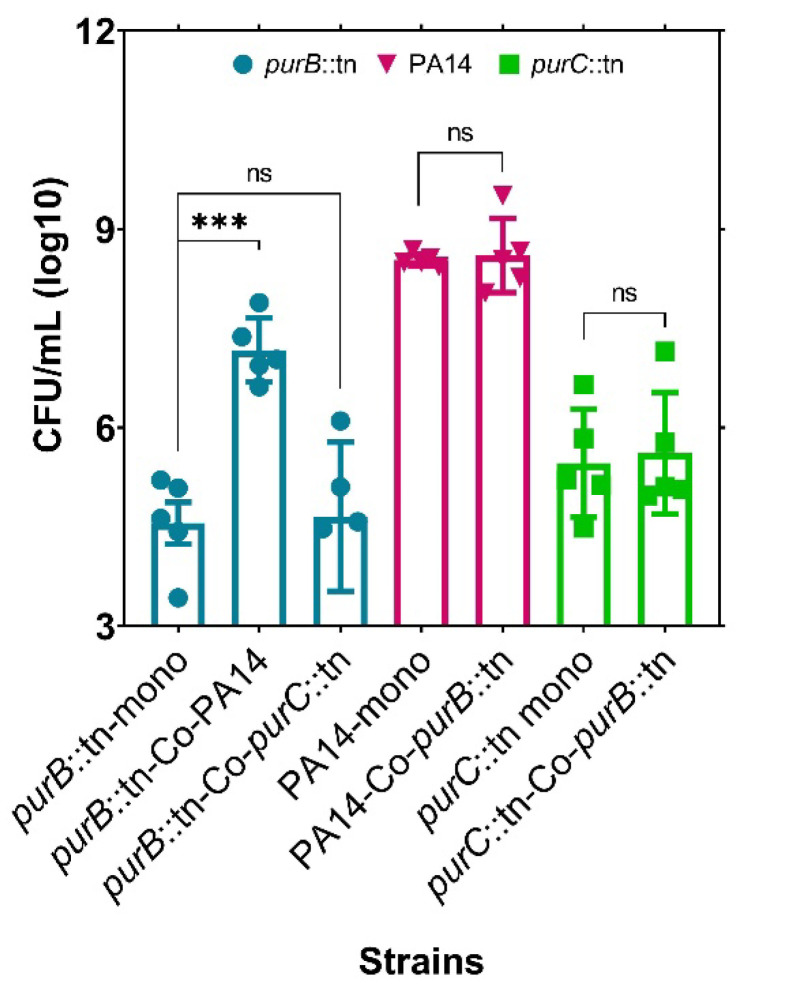
Purine complementation in purine mutant, *purB*::tn of *S. aureus*, by PA14 in co-culture. Growth of a purine-deficient mutant of *S. aureus* can be rescued by the wild-type *P. aeruginosa* cells but not by a *P. aeruginosa* purine-deficient mutant. The *purB*::tn mutant growth cannot be recovered when grown with a purine mutant of PA14, suggesting absence of cross feeding between the mutant species. Error bars represent SEM of data derived from five biological replicates on different days, and experiments were performed in technical triplicates each day. Here, ‘***’ designates *p* < 0.0005 as depicted by two-tailed unpaired Student’s *t*-test and ns denotes not significant.

To further validate the source of purine for *purC*::tn rescue, we next co-cultured this strain with a purine-deficient mutant of *S. aureus* (*purB*::tn). In this co-culture, *purB*::tn could not rescue the growth of *purC*::tn, suggesting that purines were not supplemented by *purB*::tn as it is also defective for purine biosynthesis (Figure 4). This result supports purine complementation in *purC*::tn by JE2, which possesses the intact machinery for purine biosynthesis. Interestingly, when the *purB*::tn *S. aureus* strain was co-cultured with wild-type *P. aeruginosa* strain, this *purB*::tn mutant was actually rescued by PA14 rather than being outcompeted. Only at time points exceeding 3 days was the antistaphylococcal effect of PA14 observed against the purine defective mutant of *S. aureus* (data not shown). This transition of the typical PA14 competitive behavior to slightly more cooperative behavior in the presence of *S. aureus* is interesting and requires further investigation. However, since no reduction of PA14 cell density was observed in the *purB*::tn co-culture, we believe that this growth rescue is indeed occurring via interspecies exploitation of the natural metabolism of PA14 rather than competitive lysis of PA14 by *S. aureus*.

## 3. Materials and Methods

### 3.1. Chemicals

All chemicals were purchased through Fisher Scientific unless specified otherwise. Primers for strain confirmation were purchased through Integrated DNA Technologies.

### 3.2. Bacterial Strains

The transposon mutant strain of *P. aeruginosa* and corresponding parental strain was obtained from a non-redundant library of PA14 transposon mutants, and the insertion mutants were made by using the transposon MAR2xT7 [31]. The transposon mutant strain of *S. aureus* and its corresponding parental strain were obtained from the Nebraska Transposon Mutant library, where they used USA300 LAC as the parent strain and mariner Tn *bursa aurealis* for transposon insertion [32]. Transposon mutant identity was confirmed using arbitrary PCR with the primer sets recommended by the original library creators [31,32] followed by Sanger sequencing.

### 3.3. Co-Culture in Purine-Deficient Growth Medium

Prior to inoculation of cultures into purine-deficient growth medium, the laboratory reference strain of *P. aeruginosa* UCBPP-PA14 and a transposon mutant strain, *purC*::tn, of *P. aeruginosa* were cultured in Lysogeny broth (LB), and the wild-type strain of *S. aureus*, USA300 JE2, and a transposon mutant strain, *purB*::tn *S. aureus*, were cultured in Tryptic soy broth (TSB). Overnight cultures were washed thrice with filter-sterilized 1× phosphate-buffered saline (PBS) to removed media contamination from either LB or TSB. Following washing, cells were normalized to an OD600 of 1.0 (cell density of ~10^8^). Normalized cells were diluted 100 times either as a monoculture or as co-culture in Roswell Park Memorial Institute (RPMI) medium supplemented with 1% casamino acids, which made the cell density of each strain ~10^6^ in the initial inoculum. Cells were then incubated as monocultures or co-cultures for 48 h at 37 °C under static conditions. Following incubation, bacterial cells were diluted in sterile, 1× PBS and plated on selective media; *P. aeruginosa* monocultures were plated on cetrimide agar plates, *S. aureus* monocultures were plated on mannitol salt agar plates, and co-cultures were plated on both plates to observe differences in microbial growth.

### 3.4. Purine Complementation of P. aeruginosa by Exogenous DNA

To evaluate the effect of exogenous purines in complementing deficient mutant strains, we used herring sperm DNA as an exogenous source of purine. Overnight grown cultures were washed thrice with filter-sterilized 1× PBS and then normalized to an OD600 of 1.0. Normalized cells were diluted 100 times in RPMI media supplemented with 1% casamino acids as well as 0, 10, 100, 300, 600, and 900 µg/mL of herring sperm DNA enzymatically digested with DNase enzyme at 1× for 1 h at 37 °C. These cells were then incubated in presence or absence of herring DNA for 48 h at 37 °C under static conditions. Another set of normalized cells was diluted 100 times in RPMI media supplemented with 1 % casamino acids as well as 0, 10, 100, 300, 600, and 900 µg/mL of herring sperm DNA without enzymatic digestion and incubated as mentioned before. Following incubation, bacterial cells were diluted in sterile, 1× PBS and plated on selective media agar to observe growth.

### 3.5. Measuring the Concentration of Exogenous DNA in Bacterial Culture

To measure the concentration of exogenous DNA in the bacterial culture during purine cross feeding, we co-cultured wild-type *P. aeruginosa*, PA14, and purine-deficient mutant of *P. aeruginosa*, *purC*::tn, with wild-type *S. aureus*, JE2. These bacterial strains were grown as monocultures as well. RPMI supplemented with 1% casamino acids was used as the culture medium. Following 48 h of incubation at 37 °C and at static concentration, the concentration of the eDNA in the media was measured using the Quant-iT™ PicoGreen™ dsDNA Reagent (Invitrogen, CA, USA) by following the manufacturer’s protocol with a slight modification. Briefly, samples were mixed with 200 times diluted reagents in a ratio of 1:1 in a 96-well plate. Post 5-min incubation of cultures with the reagent, at room temperature, fluorescent intensity was measured using a plate reader (BioTek Synergy H1) with an excitation of 485 nm and emission of 528 nm. The concentration of eDNA in bacterial cultures was calculated using a standard curve created from the respective bacterial monocultures grown in presence of eDNA, with concentrations ranging from 1 to 900 µg/mL.

## 4. Conclusions

**Figure 5:**
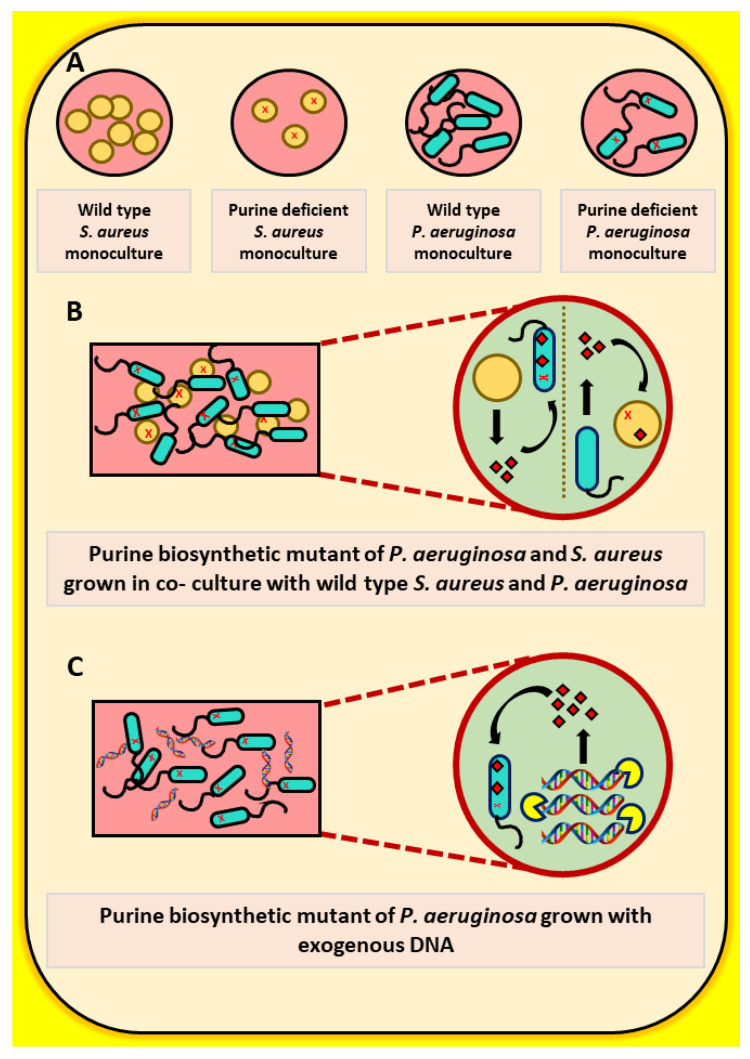
Cross feeding of purines by wild-type *P. aeruginosa* or *S. aureus* and exogenous DNA. (**A**) Monocultures of wild-type as well as purine-deficient mutants of *P. aeruginosa* and *S. aureus*. (**B**) Cross feeding of purines to *P. aeruginosa* and *S. aureus* purine biosynthetic mutants by wild-type *S. aureus* and *P. aeruginosa* cells, respectively. (**C**) Rescue in growth of *P. aeruginosa* purine biosynthetic mutants by exogenous DNA.

Overall, the data presented herein indicate that purine auxotrophies in both *P. aeruginosa* and *S. aureus* can be rescued via interspecies metabolic exchange and that this metabolic exchange may in part be mediated by natural release of eDNA rather than by competitive cell lysis (Figure 5). As purines and other metabolites have been shown to have limited bioavailability in the host CF environment [11], natural auxotrophies arising in some of these pathways during infection may instead be indicators of intra- and interspecies metabolic exchange during infection. This type of metabolic complementation could represent the first step in the evolution of cooperative/synergist interactions within polymicrobial communities [29]. Such interactions can have severe impacts during infection as polymicrobial synergy has been shown to increase antibiotic resistance and disease severity in certain cases [30]. Therefore, these types of interactions in the context of isolates from CF lungs and other chronic infections should be the focus of future studies.

## Data Availability

All raw data will be available and provided upon request.
